# Implantation Mycoses and Invasive Fungal Infections with Cutaneous Involvement in Tropical Taiwan: An 11-Year Retrospective Study of a Medical Center

**DOI:** 10.3390/jof9030322

**Published:** 2023-03-05

**Authors:** Ting-Jung Hsu, Chih-Hung Lee

**Affiliations:** 1 Department of Dermatology, Kaohsiung Chang Gung Memorial Hospital, Chang Gung University College of Medicine, Kaohsiung 833, Taiwan; 2 Institute of Translational Research in Biomedicine, Kaohsiung Chang Gung Memorial Hospital, Kaohsiung 833, Taiwan

**Keywords:** implantation mycoses, invasive fungal infections, deep fungal infections

## Abstract

Background: The rising incidence of implantation mycoses and invasive fungal infections prompts the need for studies describing the latest trends of these diseases; however, the literature remains scarce from tropical Asia in recent years. We shared our 11-year clinical experience at a tertiary center in Southern Taiwan to improve physicians’ understanding of the diseases, which could help them assume appropriate management strategies. Patients and methods: Forty cases of pathology-proven cases of implantation mycoses and invasive fungal infections with cutaneous involvement were retrospectively reviewed. The epidemiology, patients’ characteristics, initial clinical impressions, fungal species, management, and outcomes were compared and reported. Results: *Fonsecaea* sp. was the most commonly (14%) involved species in implantation mycoses. The percentages of immunocompromised patients with implantation mycoses and invasive fungal infections were 26% and 60%, respectively. Additionally, 46% of patients with implantation mycoses had type 2 diabetes mellitus. The lesions were commonly mistaken for skin appendage tumors, skin cancers, and hyperkeratotic dermatoses. The prognosis was favorable for the implantation mycoses (83% showed clinical improvement) but bleak for the invasive fungal infections (100% mortality). Conclusions: Presentations of implantation mycoses and invasive fungal infections vary widely, and immunocompromised status and diabetes mellitus are important associated factors.

## 1. Introduction

Fungal infections are some of the most common diseases in dermatology, affecting a billion patients globally [1]. Fungal infections can be broadly classified by the depth of involvement. Superficial fungal infections, such as dermatophytosis and cutaneous candidiasis, affect the epidermis and adnexal structures, whereas implantation mycoses, also known as subcutaneous fungal infections, involve the dermis and subcutaneous tissues. Invasive fungal infections refer to systemic conditions that involve internal organs, yet these diseases can also manifest as cutaneous lesions when the skin is affected. 

While superficial fungal infections can be easily treated, the implantation mycoses and invasive fungal infections can cause significant morbidity and mortality [1,2]. The cutaneous manifestations of implantation mycoses and invasive fungal infections with skin involvement are diverse, and a skin biopsy is usually needed to confirm the diagnosis and identify causative organisms [3,4]. The diagnosis remains a big challenge due to the non-specific skin findings [5] and the difficulty of a fungal culture [6]. A recent rise in incidence [7] has revealed a more imminent necessity for both general practitioners and dermatologists to be familiarized with the clinical manifestations and management of the diseases. Despite a few studies that have summarized cases from the first decade of the 21st century [8,9], the literature in recent years remains scarce for better characterizing the latest increase of deep fungal infections in Asia, particularly in tropical Asia. 

This study aimed to broaden the understanding of the increasing implantation mycoses and invasive fungal infections with cutaneous involvement by summarizing the recent 11-year experience at a tertiary medical referral center in Southern Taiwan.

## 2. Patients and Methods

### 2.1. Study Site

This study was conducted at Kaohsiung Chang Gung Memorial Hospital, a tertiary medical center in tropical Taiwan (geolocalization: 22°39′02″ N, 120°21′22″ E).

### 2.2. Study Design and Population

From 2012 to 2022, all the medical records of patients visiting the study site for the suspicion of fungal infection were considered. Included patients were those with diagnoses of skin fungal infections that were proven by pathological examination of the biopsied tissues that did or did not have fungal culture results. The patients with known superficial fungal infections, fungal colonization in keratin layers, or fungal folliculitis were not included.

### 2.3. Case Definition and Studied Variables

The patients were classified as having implantation mycoses if no evidence of systemic involvement was found, or as having invasive fungal infections otherwise. The clinical records were manually reviewed by board-certified dermatologists to obtain information regarding the age, gender, risk factors including gardening habits and immunosuppression status, clinical presentations, first clinical impressions, pathology findings, culture results, antigen research, underlying systemic diseases, disease duration, treatment, and outcomes.

### 2.4. Statistical Analysis

Descriptive statistics were calculated for quantitative variables, including the median and range of age and the median duration of lesions. Frequencies were calculated for the following qualitative variables: gender, history of local injury, gardening habits, immunocompromised state, type 2 diabetes mellitus, first impression, obtaining fungal culture, and improvement after treatment.

## 3. Results

A total of 65 medical records were reviewed, and 40 cases were included. Among the 40 patients, 35 patients had implantation mycoses, and 5 patients had invasive fungal infections with a cutaneous involvement. Table 1 summarizes the patient characteristics for both groups.

### 3.1. Patient Characteristics

Male patients were more commonly involved than female patients in both types. The median age in years was 68 and 64 for implantation mycoses and invasive fungal infections, respectively. Nine patients (26%) with implantation mycoses were immunocompromised. Included in these cases, one had acute myeloid leukemia (AML) and was on palliative chemotherapy, one had lymphoma and was receiving chemotherapy, one had acquired immune deficiency syndrome with low CD4 cell count, and six patients were receiving long-term (>1 month) systemic corticosteroid treatment for systemic lupus erythematosus (one case), polymyositis (one case), rheumatoid arthritis (two cases), chronic obstructive pulmonary disease (one case), and autoimmune hemolytic anemia (one case). Type 2 diabetes mellitus (DM) was found in 16 patients (46%). Most patients relied on oral hypoglycemic agents, whereas one patient required insulin injections for glycemic control. The mean level of glycosylated hemoglobin (HbA1c) was 8.4% in nine patients with available laboratory results at the time of diagnosis.

Three patients with invasive fungal infections had compromised immune systems, including two patients with severe aplastic anemia and one patient with AML for which palliative chemotherapy was given. The blood counts of these patients showed severe leukopenia of less than 500 white blood cells/μL. Another patient with systemic infections had poorly controlled type 2 DM (HbA1c 10.1% at the time of infection). One patient on oral hypoglycemic agents had type 2 DM with an HbA1c of 7.1% at the time of diagnosis, but the patient did not otherwise exhibit evidence of immunosuppression.

### 3.2. Clinical Presentation and History

**Figure 1:**
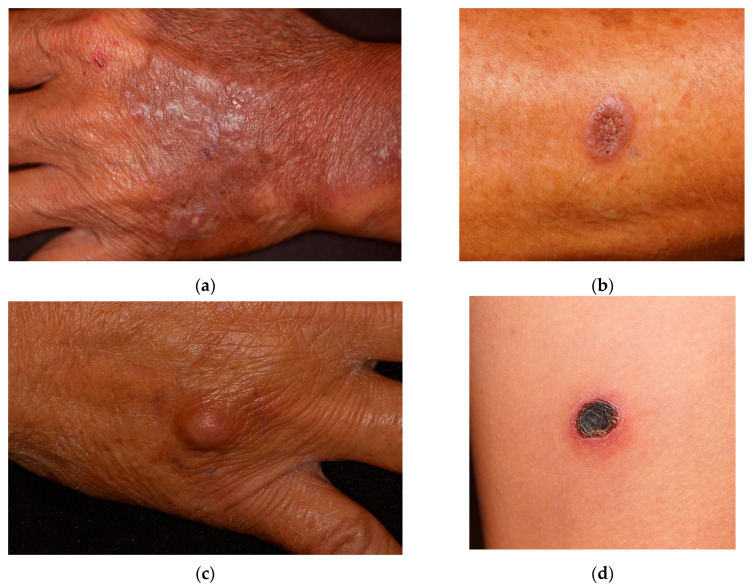
Common appearances of implantation mycoses: (**a**) papuloplaques on the dorsal hand (*Mucor* sp.); (**b**) papuloplaque with a verrucous surface (*Nigrospora*); (**c**) nodule on the dorsal hand (unknown pathogen); and (**d**) ulceration on posterior thigh (unknown pathogen).

Implantation mycoses manifested as papuloplaques (29 cases, 83%), nodules (4 cases, 11%), and ulcerations (2 cases, 6%). The appearance of common skin lesions is shown in Figure 1. In four cases (11%), the lesions developed bilaterally. The onset was usually slow, with a median duration of 5 months. There were 21 patients (60%) who were asymptomatic; other reported accompanying symptoms were mostly mild, including pain in 7 patients, itchiness in 6 patients, and a burning sensation in 1 patient. Common locations were the upper limbs, especially the forearms or dorsal hands (26 patients, 74%), with fewer patients developing lesions on the lower limbs (4 patients, 11%) and posterior thighs (2 patients, 6%). Other patients presented lesions on the upper and lower extremities (one patient, 3%), abdomen (one patient, 3%), and eyelid (one patient, 3%).

**Figure 2:**
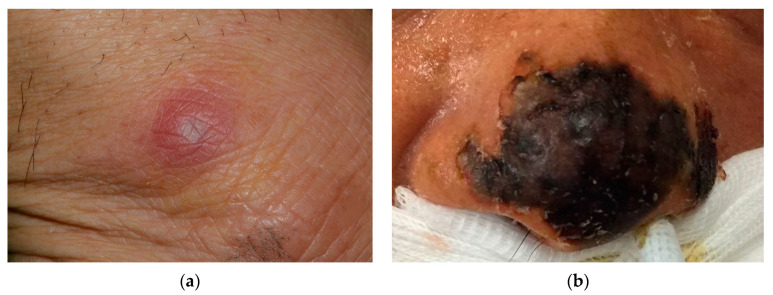
Skin lesions of systemic mycoses: (**a**) erythematous papules on the ankle and four limbs (unknown pathogen); and (**b**) necrotic plaques on the nose tip (*Candida albicans*).

The skin lesions of the invasive fungal infections usually developed acutely with bilateral involvement within a few days. The most common presentation was multiple erythematous papules on the bilateral upper and lower limbs with or without tenderness, seen in three patients (Figure 2a). In the other two patients, necrotic plaques on the noses were the initial presentation (Figure 2b), and one of them also developed multiple purpuric patches on bilateral distal limbs.

The history of contact with plants or crops was more commonly elicited in patients with implantation mycoses. Eight patients (23%) with local infections reported gardening habits. Three patients recalled a history of injury or penetrating injury by plants before the infections ensued. In contrast, none of the patients with invasive fungal infections reported agricultural injury or a history of gardening work. None of the patients with invasive fungal infections had prior superficial fungal infections according to medical records.

### 3.3. First Clinical Impression before Biopsy

The physicians correctly concluded subcutaneous mycoses as the first differential diagnosis in 24 patients (69%). The common differential diagnoses for cases in which the subcutaneous mycoses were not suspected were skin cancers (two cases, 6%), epidermal cysts (two cases, 6%), and ganglion cysts (two cases, 6%), followed by granuloma annulare, foreign body granuloma, lipoma, impetigo, and porokeratosis (each with one case). Among cases with correct impressions, fungal culture from biopsied tissues was performed in 75% of patients, whereas only 36% of patients of incorrect first impressions had fungal cultures.

### 3.4. Histopathological Examination

Periodic acid–Schiff (PAS) and Grocott’s methenamine silver (GMS) staining showed positive fungal evidence in 28 (80%) and 3 (60%) patients in implantation mycoses and invasive fungal infections, respectively. Most pathology findings presented with granulomatous inflammation or microabscess. Chromoblastomycosis was found in seven cases (20%), as evidenced by brownish muriform cells and pseudoepitheliomatous hyperplasia microscopically. None of the patients had phaeohyphomycosis.

### 3.5. Microbiology

Out of 35 cases of implantation mycoses, fungal cultures from the skin tissue were performed in 22 patients (63%). Among these obtained cultures, no fungal growth was found in eight cases (36%). Among the cultures showing positive fungal growth, the most commonly identified pathogens were *Fonsecaea* sp. in five cases (14%), followed by non-specific molds in three cases, *Candida albicans* in one case, and one case each for *Prototheca* sp., *Nigrospora* sp., *Aspergillus niger*, *Mucor* sp., and *Microsphaeropsis arundinis*. Two patients had fungal growth from the aerobic bacterial culture of pus, which showed mold and *Pleosporales*.

Four patients with invasive fungal infections underwent fungal cultures from skin tissue. *Candida albicans* was identified in one patient, *Fusarium* sp. was found in another patient, and no fungal growth was noticed in the other two. One patient underwent an aerobic bacterial culture, which later showed the growth of molds.

### 3.6. Treatment

The most commonly prescribed first-line treatment for the implantation mycoses in the studied patients was oral itraconazole, which was used in 25 patients (71%). Other antifungal agents of choice included fluconazole (two cases, 6%) and terbinafine (one case, 3%). The median itraconazole treatment duration was 12 weeks. Itraconazole was used as a single treatment if clinical improvement was observed, or switched to other antifungal medications if the treatment response was poor, which was noticed in eight patients. Three patients had mildly elevated liver function (elevation of alanine transaminase within 3 times the normal upper limits) after taking oral itraconazole; however, the medications were continued unless a poor clinical response was noted. In the 15 patients (43%) using itraconazole as a single regimen, 10 patients showed an obvious clinical improvement (the lesions shrunk in size, and the patient discontinued oral medication). Among the 28 patients (80%) that were administered the oral antifungal agents, five patients (18%) underwent subsequent surgical excisions of skin lesions after the lesions became sufficiently small for surgery. Seven patients (20%) underwent total excisions of skin lesions directly without prior oral medications, and two of them were given 2 weeks and 5 weeks of itraconazole after the surgery, respectively.

Voriconazole was used as the initial antifungal agent in three patients with systemic infections. The other two patients received systemic micafungin and amphotericin B, respectively.

### 3.7. Clinical Outcomes

Twenty-nine patients (83%) with implantation mycoses improved after an antifungal treatment and/or surgery. No implantation mycoses-related mortality was noticed. Three cases had a disease recurrence after an initial clinical improvement. All patients with invasive fungal infections showed no improvement after systemic antifungal treatments and died from the infections within days to weeks of the diagnosis.

## 4. Discussion

In this study, we reviewed 40 cases of implantation mycoses and invasive fungal infections with a cutaneous involvement across an 11-year span at a tertiary medical center in Southern Taiwan; we summarized the initial clinical presentations, first clinical impressions from dermatologists, microbiology, pathological findings, treatment, and the clinical outcomes of these diseases. The characterization of the recent trends in the increasing incidence of these diseases indicates that physicians should perform necessary evaluations and administer appropriate treatments.

The findings suggest that *Fonsecaea* sp. was the most common species involved in implantation mycoses. The high incidence of *Fonsecaea* sp. was also discovered in other previous studies from Taiwan [8] and countries in South Asia, including India [5] and Sri Lanka [10]. However, the distribution of the pathogen species exhibited a remarkable geographical variation. For example, in a 12-year retrospective study in South Korea [11], *Candida* sp. was the top identified species, and *Fonsecaea* sp. was not found in any patient. In another study, which included 33 cases in the United States [12], *Blastomyces dermatitidis* was the most common species, and *Fonsecaea* sp. was again not identified. Therefore, the difference in climate and the prevalence of the local species must be considered during the clinical evaluation.

The upper extremities, especially the forearms and dorsal hands, were the most frequently infected in this study. Interestingly, this contrasts with previous studies in which the lower extremities were the most common infection sites [5,9,11,13,14]. The cause of such a difference remains unclear. However, because local infections usually originate from the direct inoculation of fungi by penetrating trauma, this may be due to the difference between gardening habits and agricultural work on lower leg protection. In fact, all eight patients with gardening histories had lesions on their forearms, supporting the hypothesis that some unnoticed minor injuries had possibly occurred. 

The first clinical impression before a biopsy varied greatly due to the non-specific presentation of the deep fungal infections and the infrequency of a reported trauma history. The difficulty of making the correct differential diagnoses led to a lower rate of performing timely and appropriate culture procedures during the evaluation. From the clinical appearances, the lesions were commonly mistaken as skin appendage tumors, hyperkeratotic lesions, and skin cancers. It is thus important for clinicians to become aware of other diagnosis clues, such as gardening or agriculture-related history and the presence of DM or immunosuppression. Only one out of 11 patients with incorrect diagnoses did not have any of the abovementioned clues, demonstrating the utility of this approach.

Several limitations exist in this study. First, the incidence of invasive fungal infections may have been underestimated because critical patients may not receive skin biopsies or fungal cultures. A prospective study that includes a biopsy or culture for all suspicious cases will be helpful in accurately estimating the incidence of invasive fungal infections. Second, species identification was not successful in many cases due to laboratory limitations. To construct a more detailed profile of species in fungal infections, future collaboration with advanced fungal laboratories will be beneficial in both clinical and research settings. Finally, the results of a single-center study also suffer from the lack of generalizability to other geographical areas. Careful interpretation must be taken when seeing patients in other regions.

## 5. Conclusions

*Fonsecaea* sp. are commonly identified causative pathogens in implantation mycoses. Implantation mycoses and invasive fungal infections could present in a variety of ways and are associated with immunocompromised status and diabetes mellitus.

## Figures and Tables

**Table 1:** The demographics and clinical characteristics in implantation mycoses and invasive fungal infections with skin involvement.

	Implantation Mycoses (n = 35)	Invasive Fungal Infections with Skin Involvement (n = 5)
**Patient characteristics**		
Median age (range)	68 years (9–88)	64 years (50–71)
Male/female ratio	25:10	4:1
History of local injury caused by plants	3 (9%)	0 (0%)
Gardening habits	8 (23%)	0 (0%)
Immunocompromised	9 (26%)	3 (60%)
Hematologic malignancy under chemotherapy	2	1
Aplastic anemia	0	2
Acquired immunodeficiency syndrome	1	0
Systemic corticosteroid > 1 month	6	0
Type 2 diabetes mellitus	16 (46%)	2 (40%)
**Clinical characteristics**		
Median duration of lesions	5 months	3 days
Correct first impression ^1^	24 (69%)	4 (80%)
Fungal culture performed	22 (63%)	4 (80%)
Improvement after antifungal treatment ^2^	29 (83%)	0 (0%)

^1^ Fungal infection was listed as one of the differential diagnoses after the first evaluation. ^2^ Lesions shrunk in size.

## Data Availability

The data is unavailable due to patient privacy.
